# Magnetic Control of Magneto-Electrochemical Cell and Electric Double Layer Transistor

**DOI:** 10.1038/s41598-017-11114-2

**Published:** 2017-09-05

**Authors:** Takashi Tsuchiya, Masataka Imura, Yasuo Koide, Kazuya Terabe

**Affiliations:** 1 International Center for Materials Nanoarchitectonics (WPI-MANA), National Institute for Materials Science (NIMS), 1-1 Namiki, Tsukuba, Ibaraki 305-0044 Japan; 2 Research Center for Functional Materials, NIMS, 1-1 Namiki, Tsukuba, Ibaraki 305-0044 Japan; 3 Research Network and Facility Services Division, NIMS, 1-2-1 Sengen, Tsukuba, Ibaraki 305-0047 Japan

## Abstract

A magneto-electrochemical cell and an electric double layer transistor (EDLT), each containing diluted [Bmim]FeCl_4_ solution, have been controlled by applying a magnetic field in contrast to the control of conventional field effect devices by an applied electric field. A magnetic field of several hundred mT generated by a small neodymium magnet is sufficient to operate magneto-electrochemical cells, which generate an electromotive force of 130 mV at maximum. An EDLT composed of hydrogen-terminated diamond was also operated by applying a magnetic field. Although it showed reversible drain current modulation with a magnetoresistance effect of 503%, it is not yet advantageous for practical application. Magnetic control has unique and interesting characteristics that are advantageous for remote control of electrochemical behavior, the application for which conventional electrochemical devices are not well suited. Magnetic control is opening a door to new applications of electrochemical devices and related technologies.

## Introduction

Electrochemistry has been playing a leading role in myriad applications, ranging from energy and environmental technologies (e.g., batteries, capacitors, sensors) to information and communication technologies (e.g., resistive memory devices)^1–14^. Useful functions for such applications originate from ionic transport in the electrolyte, accompanied by electrochemical processes near the electrode/electrolyte interface, electric double layer (EDL) generation, and electrochemical reduction and oxidation (redox). Control of the ionic transport is particularly important for various electrochemical devices including EDL devices [e.g., EDL capacitors, EDL transistors (EDLTs)]^1–12^ and redox devices (e.g., batteries, resistive memory devices)^13, 14^.

Conventional electrochemical devices are usually operated by applying an external electric field through conductive electrodes attached to the device. An external electric field effectively controls the ions, which have positive or negative charges. Electrical control of ionic transport is particularly useful due to the abundance of electricity in modern society and its precise controllability.

While the electric control of ionic transport has been exploited for most electrochemical devices, it intrinsically limits electrochemical devices, including device structure and materials selection^13^. Structural design is limited by the use of electrodes to connect to a voltage source. Furthermore, the electrode materials must be relatively conductive. This makes application of resistive materials to electrochemical devices difficult. As long as the conventional operation principle based on electric field control of ionic transport is used, these limitations cannot be overcome. However, if another external field could be used to drive the ions, novel electrochemical devices free from conventional limitations might be achievable.

We have demonstrated magnetic control of a magneto-electrochemical cell and an EDLT, each containing diluted [Bmim]FeCl_4_ electrolyte, by application of a magnetic field instead of an electric field. Operation of these devices is achieved using a unique and excellent transport property of FeCl_4_^−^ ions under a magnetic field^15^. A magnetic field of several hundred mT generated by a small neodymium magnet enabled generation of an electromotive force of 130 mV at maximum in magneto-electrochemical cells (MECs). Such magnetic control was used to operate an EDLT, and magnetic-field-induced drain current modulation with a huge magnetoresistance (MR) effect was demonstrated using an EDLT with hydrogen-terminated diamond epitaxial thin film as a channel material. Although this technique does not provide a significant advantage due to serious problems at present (*e*.*g*., energy consumption for magnetic field generation, use of liquid electrolyte, use of permanent magnet), this magnetic control technique offers a new approach to electrochemical device development. For example, it could be used to remotely control electrochemical devices, the application for which conventional electrochemical devices are not well suited.

## Experimental Setup and Optical Characterization of Magneto-Electrochemical Cell

**Figure 1:**
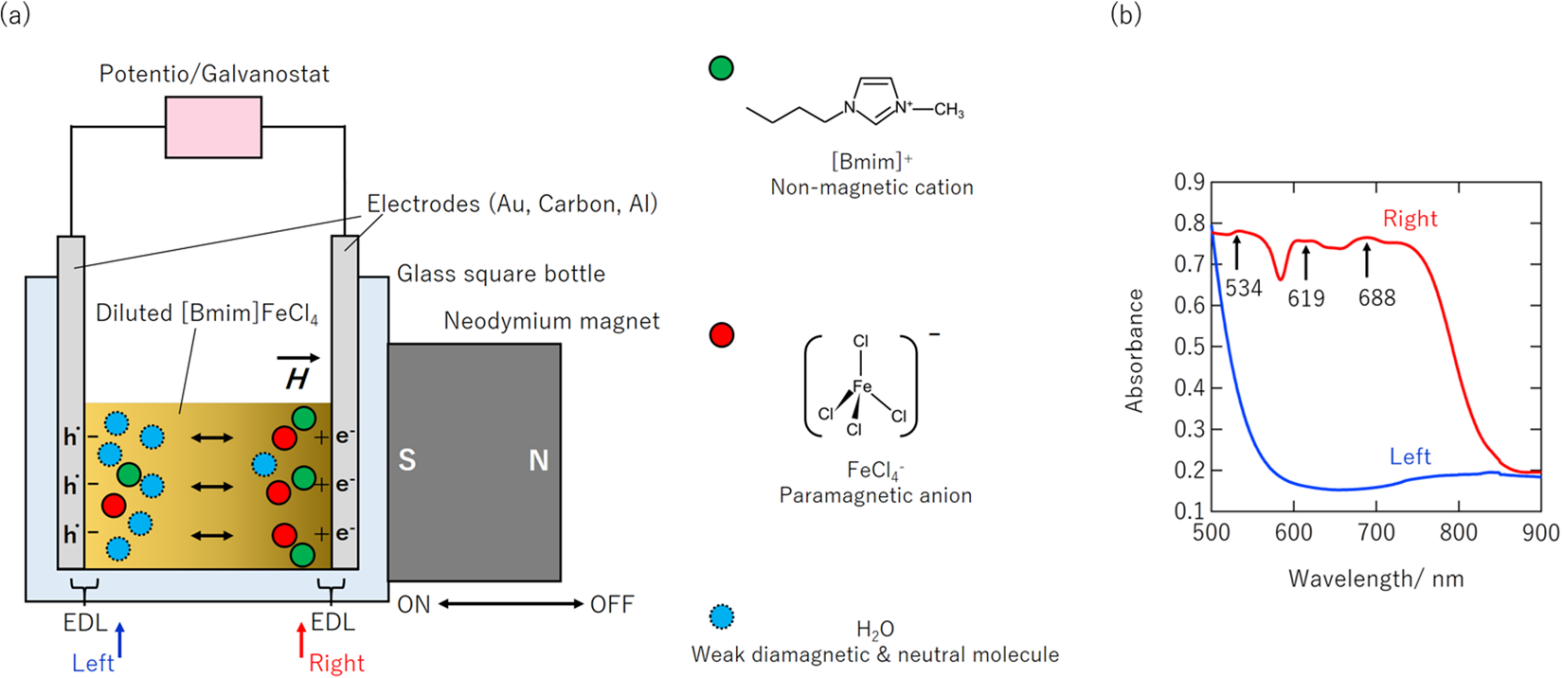
(**a**) Illustration of two-terminal electrochemical-cell-type MEC and components of diluted [Bmim]FeCl_4_ solution. (**b**) UV-Vis-NIR absorption spectra measured near right electrode (dark brown) and near left electrode (light yellow).

Figure 1(a) shows a schematic illustration of the two-terminal magneto-electrochemical cell (MEC) used in this study. Two Au electrodes are placed on the left and right sides of a glass square bottle filled with [Bmim]FeCl_4_ liquid electrolyte diluted by distilled water to the desired concentration from 100% (pure [Bmim]FeCl_4_) to 10%. The electrical properties of the MEC were investigated using a potentio/galvanostat (Compactstat, Ivium Technologies, Italy) by attaching or removing small neodymium magnets possessing various magnetic flux densities in order to switch the magnetic field on and off. A magnet was attached to the right or left side to apply a magnetic field to the MEC. The force (*F*) applied to a magnetic dipole moment (FeCl_4_^−^ ion) can be described by

1 $$F=(1/{\mu }_{0})\chi H\frac{\partial {H}_{x}}{\partial x}$$

where *μ*_0_ and *χ* are magnetic permeability of vacuum and magnetic susceptibility of FeCl_4_^−^ ions. The sign of *χ* is positive due to due to the paramagnetic characteristics. The signs of *H* and magnetic field gradient, $\frac{\partial {H}_{x}}{\partial x}$, are opposite each other in the experimental conditions. Therefore, *F* always takes a negative value, corresponding to the attraction toward a magnet. This means that *H* switching from positive to negative has no effect on the transport behaviour of FeCl_4_^−^ ions although the absolute values of *H* and $\frac{\partial {H}_{x}}{\partial x}$ have a substantial effect according to eq. (1). See Supplement 1 (S1) for discussion on the magnetic field and magnetic field gradient profiles calculated for the MEC using various magnets. *H* switching (*H* reversal) should also switch the directions of the magnetic moments of ions, but this reversal can affect the locations of the surrounding non-magnetic ions involved in the formation of EDL and corresponding electrostatic potential near the interface. This effect was investigated in additional experiment using *H* application by S polar or N polar of permanent magnet (see S2).

All of the experiments were performed in air at room temperature. See the Methods section for the experimental details.

When a magnetic field of 480 mT was applied to the MEC from the right side, dark brown and light yellow regions were evident near the right and left electrodes. They indicated a high concentration and a low concentration of [Bmim]FeCl_4_ liquid electrolyte, respectively. Although only the FeCl_4_^−^ ions (strongly paramagnetic) were sensitive to a magnetic field, the non-magnetic [Bmim]^+^ ions were also transported by ambipolar diffusion with the FeCl_4_^−^ ions, resulting in a [Bmim]FeCl_4_ concentration difference (see S1). This means that the magnetic-field-induced diffusion of ions overcomes their thermal diffusion in a magnetic field of 480 mT.

Figure 1(b) shows the UV-Vis-NIR absorption spectra near the right electrode (dark brown) and near the left electrode (light yellow). The three absorption features located at 534, 619, and 688 cm^−1^ in the spectrum near the right electrode are attributed to molecular absorption of the FeCl_4_^−^ ions in the [Bmim]FeCl_4_^16^. The very weak absorption of the features in the spectrum near the left electrode indicates a significant difference in the [Bmim]FeCl_4_ concentration between the two electrodes, which offers unique and useful electromotive force variation, as discussed below.

## Results and Discussion

### Electromotive force observation with magnetic field switching

**Figure 2:**
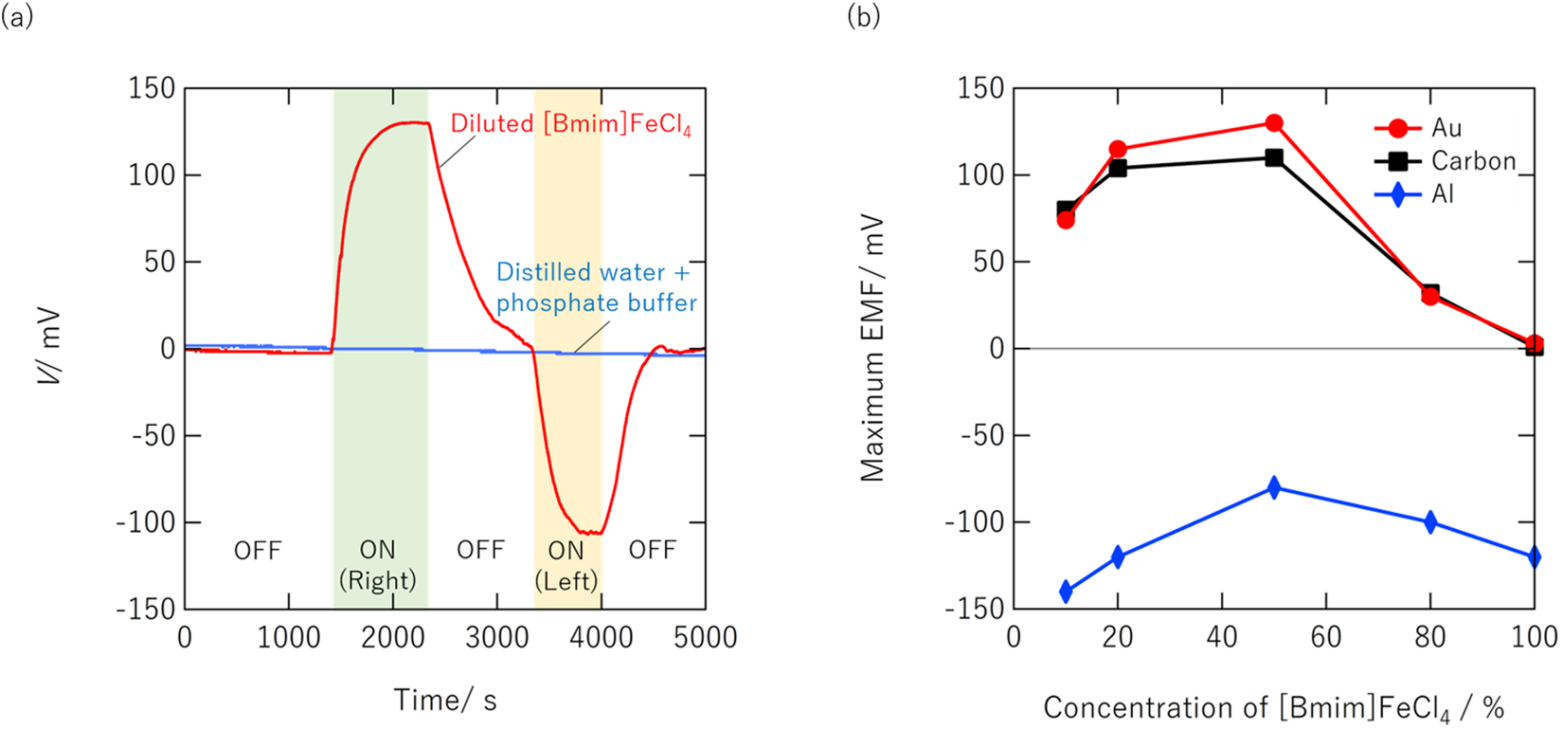
(**a**) Variation in OCV of MEC composed of two Au electrodes (Au/Au) and 50% diluted [Bmim]FeCl_4_ solution. (**b**) Maximum EMF dependence on [Bmim]FeCl_4_ concentration of MECs composed of various electrode materials.

Figure 2(a) shows the variation in the open circuit voltage (OCV) of an MEC with two Au electrodes (Au/Au) and 50% diluted [Bmim]FeCl_4_ solution. Voltage is defined here as *E*_left_ − *E*_right_, where the electrode potentials of the left and right electrodes are *E*_left_ and *E*_right_, respectively. Voltage thus takes a positive value when *E*_left_ is higher than *E*_right_. When a magnetic field was applied to the MEC from the right side, the OCV immediately started to increase and reached 130 mV. The applied magnetic field made the [Bmim]FeCl_4_ concentration near the right Au electrode much higher than that near the left, causing a significant electromotive force (EMF) between the two electrodes. In other words, the EDLs near the two Au electrodes were differently charged by the magnetic field.

The magnetic field causes a gradient of FeCl_4_^−^ ions that overcomes the thermal diffusion. This generates an electric field near the electrodes that charges the EDLs. This corresponds to the illustration shown in Fig. 1(a). The positive OCV returned to zero when the magnetic field was removed. When a magnetic field was applied from the left side, the EMF moved in the opposite direction (negative). This symmetric OCV behaviour clearly demonstrates that the EMF observed in the OCV originated from the strong attraction of [Bmim]FeCl_4_ to one side and the resultant difference between *E*_left_ and *E*_right_. This was confirmed using an MEC with a reference electrode (see S2).

The EMF is attributed to the ion concentration difference in the electrolyte; that is, the MEC works as a concentration cell, where electrode potential is determined by an equilibrium of electron and other chemical species (*e*.*g*., ions, metals) at the electrode/electrolyte interface along Nernst equation^17^. The specific arrangement of ions near the interface (i.e. surface excess of ions) and corresponding carrier type introduced at an electrode surface are determined by the thermodynamic equilibrium of electron and chemical components near the electrode/electrolyte interface.

Given that the equilibrium of ${{\rm{FeCl}}}_{4}^{-}+e^{\prime} \to {{\rm{Fe}}}^{2+}+4{{\rm{Cl}}}^{-}$, reaction (2), determines *E*_left_ and *E*_right_, the *E*_left_ and *E*_right_ for the cell can be understood on the basis of the Nernst equation:

2 $$E={E}^{0}+\frac{RT}{F}\,\mathrm{ln}\,\frac{[{{\rm{FeCl}}}_{{\rm{4}}}^{-}]}{[{{\rm{Fe}}}^{{\rm{2}}+}]\cdot {[{{\rm{Cl}}}^{-}]}^{4}}$$

where *E* ^0^, [FeCl−_4_], [Fe^2+^], and [Cl^−^] are the standard electrode potential of reaction (2) and the concentration of FeCl_4_^−^, Fe^2+^, and Cl^−^ ions, respectively. The *E*_left_ and *E*_right_ are varied in the potential range near *E* ^0^ by variation in the concentration of ions. Estimation based on a redox potential of Fe^3+^/Fe^2+^ in the Pourbaix diagram^18^ gives an *E* ^0^ close to 600 mV vs. SSE (silver/silver chloride reference electrode). The *E*_left_ and *E*_right_ were observed in the range 520 to 640 mV vs. SSE (see S2), which supports our assumption regarding the origin of the EMF.

Distilled water with a phosphate buffer (shown by the blue curve in Fig. 1(c)), which was added to obtain sufficient ionic conductivity in the distilled water, showed no EMF response to the magnetic field. This finding rules out a contribution from the weak diamagnetic property of water molecules to the OCV variation. Repeatability of the EMF modulation was demonstrated in additional experiments (see S2).

Figure 2(b) shows the dependence of the maximum EMF on the [Bmim]FeCl_4_ concentration of MECs with electrodes composed of three different materials. While the EMF of the MECs with Au/Au or carbon/carbon electrodes was almost zero for pure [Bmim]FeCl_4_, it was higher for diluted [Bmim]FeCl_4_, indicating that the EMF depends on the [Bmim]FeCl_4_ concentration difference between the electrodes. The maximum EMF was over 100 mV at 50% [Bmim]FeCl_4_ for both Au/Au and carbon/carbon while further dilution resulted in a slight decrease in the maximum EMF.

In contrast, the EMF of the MEC with Al/Al electrodes was large even for pure [Bmim]FeCl_4_, strongly indicating a different origin for the EMF. The noisy and unstable OCV variation with time (see S3) and generation of aluminium chloride on the surface of the Al electrode (see S4) indicate that the EMF was caused by the electrochemical reaction of chloride ions with the Al, which is highly active^19^. This interpretation is consistent with the finding that Au and carbon, which are relatively inert in electrochemical reactions, showed stable and completely different EMF characteristics. Although such electrochemical reactions of active electrodes controlled by switching the magnetic field could also be exploited for energy devices, we concentrated on MECs with inert electrodes in the present study. The results indicate that our MEC is not a conventional electrochemical device, which requires an external electric field.

### Discharge curves of MEC

**Figure 3:**
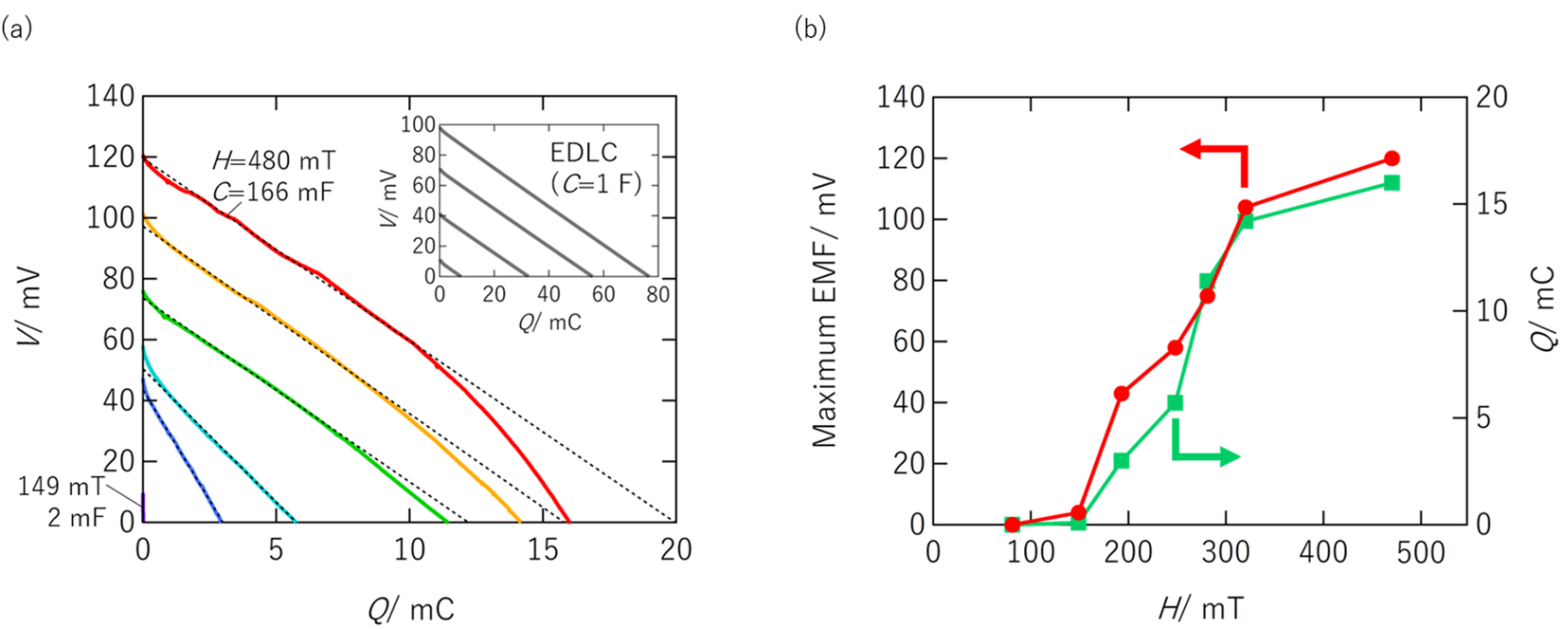
(**a**) Discharge performance (*V vs*. *Q*) of MEC with Au/Au electrodes measured under constant current condition (*i* = 1 *μ*A). Inset shows discharge performance of EDLC (*C* = 1F) for comparison. (**b**) Magnetic field dependence of maximum EMF and *Q*.

Figure 3(a) shows the discharge performance (*V vs*. total charge, *Q*, discharged from MEC) of an MEC with Au/Au electrodes measured under a constant current condition (*i* = 1 *μ* A). The slope of the *V* vs. *Q* relationship $(\frac{dV}{dQ})$ corresponds to an inverse of capacitance (1/*C*) at each *Q* or *V*. This means that the slope should be constant (*i*.*e*., the *V* vs. *Q* relationship should be on a straight line) if the device works as a capacitor. To discuss the operation mechanism of our MEC, additional lines are drawn in Fig. 3(a) by a linear approximation of the *V* vs. *Q* relationship (assuming the MEC as a capacitor) although the deviation was significant. For comparison, the *V* vs. *Q* relationship of a EDLC (*C* = 1F) is also shown in Fig. 3(a) inset. The four straight lines in Fig. 3(a) inset correspond to discharge performance of the EDLC measured under different initial charge conditions (8, 32, 56, and 77 mC from left to right, respectively). The constant slope in the *V* vs. *Q* relationship of the EDLC evidences that *C* of the EDLC is constant regardless of initial charge amount. Therefore, we can confirm that the EDLC works in a capacitor mechanism.

Based on the following reasons, we concluded that our MEC is not working in a capacitor mechanism. First, in our MEC, the deviation from the additional lines was significant as mentioned above. Second, the capacitance values of the MEC calculated from the slopes varied widely with respect to the magnetic field, in contrast to that the constant capacitance was observed in the EDLC regardless of initial charge amount. Third, the calculated capacitance for the MEC was far larger (more than a thousand times) than that expected with respect to the electrode area and specific EDL capacitance reported previously (*e*.*g*., several to several tenths *μ*F/cm^2^)^9–12^. These results indicate that the contribution of the EDL charge to the *Q* observed in the discharge was very small. This means that most of *Q* has an origin that differs from the EDL charge, although the EDL charge is modulated by the magnetic field, as will be discussed later.

Considering the strong dependence of *Q* observed in the discharge on the volume of the electrolyte (see S5), we attribute the *Q* to Faradaic charge for electrochemical reaction of the electrolyte. Possible reactions are reaction (2) and $4{{\rm{H}}}_{2}{\rm{O}}\to 4{{\rm{H}}}^{+}+{{\rm{O}}}_{2}+4{\rm{e}}^{\prime}$ [reaction (3)]. Oxidation of water molecule [reaction (3)] generates electrons which electrochemically reduce strong paramagnetic FeCl_4_^−^ to generate Fe^2+^ and Cl^−^ which are non-magnetic ions [reaction (2)].

The *Q* observed in the discharge (of the order of 10 mC) was far smaller than the total amount of FeCl_4_^−^ in the cell (equivalent to several hundred C); the fraction of FeCl_4_^−^ reduced in the discharge was very small (less than 0.01% of total FeCl_4_^−^). Therefore, the discharge finishes when the EMF (*E*_left_ − *E*_right_) becomes zero due to the concentration variation of all related ions (FeCl_4_^−^, Fe^2+^ and Cl^−^) during the electrochemical reactions.

Figure 3(b) shows the dependence of the maximum EMF and observed *Q* on the magnetic field. Both increased greatly in the magnetic field range 200–280 mT. In particular, the *Q* was two orders of magnitude smaller at 150 mT than at 280 mT. The small *Q* in a low magnetic field was due to the thermal diffusion force which is stronger than the gradient of the magnetic field in such a low magnetic field range and the resultant small [Bmim]FeCl_4_ concentration modulation near the two Au electrodes. Note that a force on magnetic moments is exerted not by uniform magnetic field, but by the magnetic field gradient. Therefore, enhancing the magnetic field gradient near the electrodes will improve MEC performance.

The interface between high and low concentration regions can cause a junction potential that completely differs from the EDL potential at the electrode interfaces. A contribution from the junction potential to the observed EMF of the MEC was measured to be approximately 2 mV, corresponding to about 3% of the observed EMF (70 mV), in additional experiment (see S6).

### Drain current modulation behaviour of magnetic field effect transistor

Two-dimensional hole gas (2DHG) near the hydrogen-terminated surface of diamond shows excellent transport properties and has thus been applied to field effect transistors (FETs) for next-generation power electronics^20–25^. The *p*-type conductivity can be electrostatically modulated not only by using conventional FETs composed of a solid dielectric but also EDLTs composed of liquid electrolytes^26–28^. In the present study, EDL control using a magnetic field was applied to carrier density modulation in the 2DHG of diamond.

**Figure 4:**
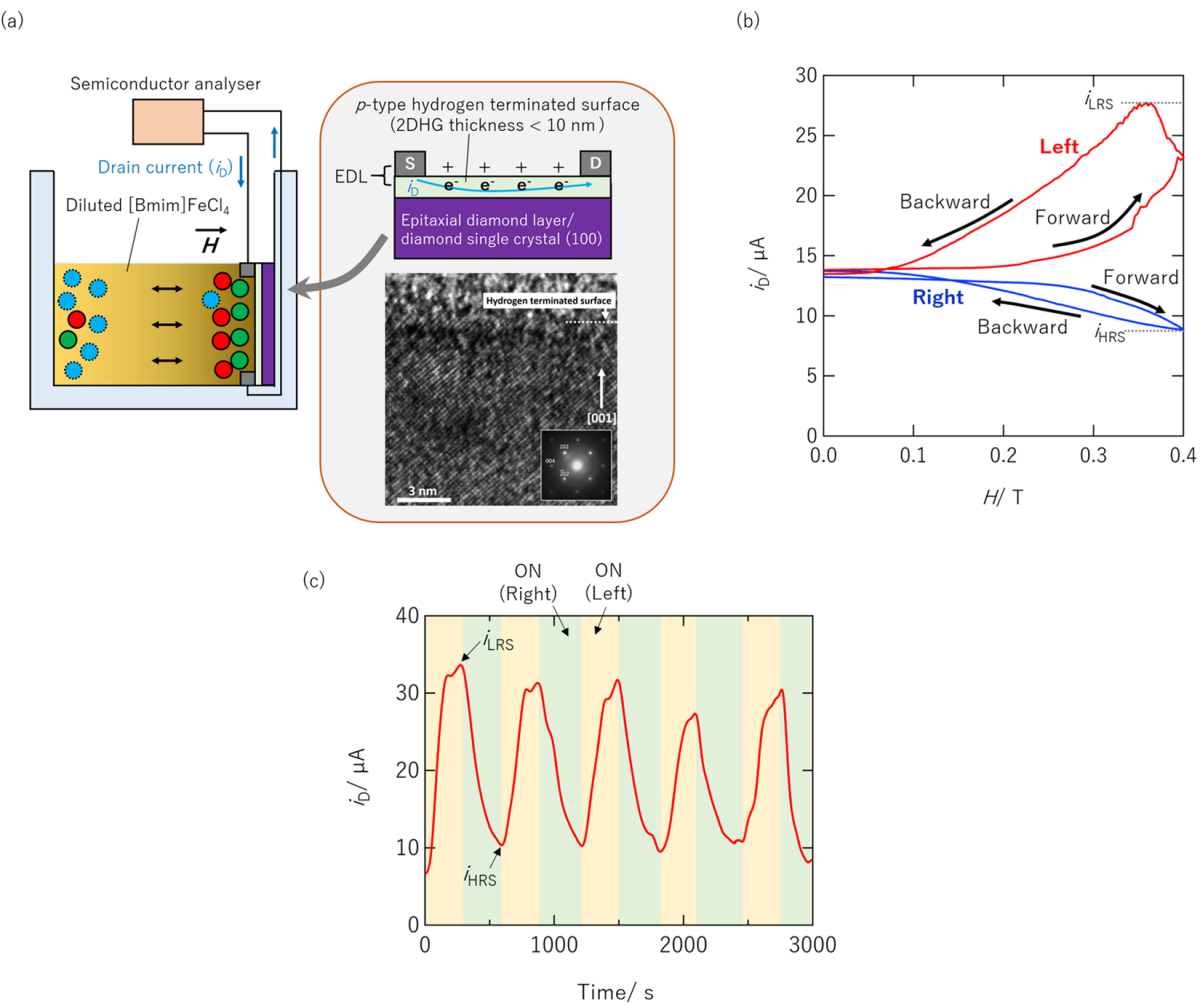
(**a**) Illustration of MFET composed of hydrogen-terminated diamond single crystal (100) and 50% diluted [Bmim]FeCl_4_ solution. (**b**) Variation in MFET *i*_D_ in response to magnetic field sweep. (**c**) Variation in MFET *i*_D_ in response to magnetic field switching (*H* = 480 mT).

Figure 4 (a) illustrates a magnetic field effect transistor (MFET) composed of hydrogen-terminated diamond single crystal (100) and 50% diluted [Bmim]FeCl_4_ solution. High-quality epitaxial growth with (100) orientation was confirmed by high resolution transmission electron microscopy (shown in Fig. 4(a)) and x-ray diffractometry (see S7). A hydrogen-terminated channel (500-*μ*m-long and 800-*μ*m-wide) was fabricated using UV-ozone treatment. The fabrication details are described in the Methods section.

Figure 4(b) shows the variation in the MFET drain current (*i*_D_) in response to a magnetic field sweep that were applied using an electromagnet from the left (red) or right (blue) side of the device shown in Fig. 4(a). As indicated by the red curve, *i*_D_ was reversibly modulated in enhancement mode (normally off) from 13.7 to 27.4 *μ*A with magnetic field application from the left. The MR effect, in which a magnetic field induces electronic conductivity modulation through various mechanisms (see S8), on the electronic current through a hydrogen-terminated diamond surface has been reported to occur at low temperature (below 2 K)^29^. However, such an MR effect was not observed for hydrogen-terminated diamond without magnetrolyte at room temperature (see S8). Therefore, the *i*_D_ variation (*i*.*e*. MR effect) shown in Fig. 4(b) is attributed not to an intrinsic MR effect but to an extrinsic MR effect due to hole concentration modulation by the MFET mechanism.

Magnetic field application from the right side caused *i*_D_ modulation in depression mode (normally on) from 13.6 to 8.8 *μ*A as indicated by the blue curve in Fig. 4(b). The difference corresponds to the attraction direction of [Bmim]FeCl_4_. Application of a magnetic field from the right caused the [Bmim]FeCl_4_ to be attracted toward the diamond channel and vice versa (see S1 for detail). Similar dependence on the attraction direction of [Bmim]FeCl_4_ was also observed for the MEC operation shown in Fig. 2. These findings agree with the paramagnetic characteristics of FeCl_4_^−^ ions.

In the MFET operation, the channel resistance increased (*i*.*e*., *i*_D_ decreased) due to magnetic field application accompanied by [Bmim]FeCl_4_ densification near the hydrogen-terminated channel. This means the [Bmim]FeCl_4_ densification and the resultant EDL modulation doped the electrons (*i*.*e*., decreased the hole concentration) in the channel. This behaviour agrees well with the EMF variation observed in the MEC shown in Fig. 2, demonstrating the EDL control mechanism in the present study, which is based on carrier density modulation on the electrode. Note that the effect of electron accumulation in the ON(Right) state was smaller than that of hole accumulation in the ON(Left) state. While this may indicate that, to some extent, mobility modulation (*i*.*e*., field effect mobility) is accompanied by carrier density modulation, quantitative discussion on the contribution only on the basis of the drain current variation is difficult at present.

Figure 4(c) shows *i*_D_ variation in the MFET in response to magnetic field switching (left/right). The *i*_D_ was reversibly modulated for 10-times switching without degradation although the switching ratio of the MFET varied between 256 and 503% cycle to cycle. The switching ratio is defined as $\frac{{i}_{{\rm{LRS}}}}{{i}_{{\rm{HRS}}}}$ 10^2^ (%), where *i*_LRS_ and *i*_HRS_ are *i*_D_ in a low resistance state (LRS) and a high resistance state (HRS), respectively. In principle, *i*_LRS_ and *i*_HRS_ in Fig. 4(c) correspond to the maximum *i*_D_ of the Fig. 4(b) red curve and the minimum *i*_D_ of the Fig. 4(b) blue curve, although they showed a slight deviation due to a difference in the applied magnetic field.

The switching ratio *(i*.*e*., MR effect) of 503% in the present study is far larger than that of spin transistors (less than 0.1%) and that of spin-torque-transfer magnetoresistive random access memories (typically 80 to 150%) at room temperature^30, 31^, although, at present, our technique is less advantageous for practical application.

Hydrogen-terminated diamond-based EDLTs composed of various liquid electrolytes have been reported^26–29^. Compared to the reported switching ratio (*e*.*g*. 10^5^–10^6^%), that of our MFET is small. However, the reported devices do not use the MR effect for switching. The relatively poor switching property of our MFET compared to other diamond-based devices is attributed to the small EMF (*e*.*g*., 130 mV at maximum) generated by the magnetic field with respect to the large gate voltage (*e*.*g*., >1 V) used in conventional EDLTs. Therefore, enhancement of the generated EMF is important for improving the switching ratio.

## Conclusion

MECs and EDLTs containing diluted [Bmim]FeCl_4_ solution have been developed. These devices operate in a magnetic field in contrast to conventional electrochemical cells and EDLTs, which need an external electric field for operation. Furthermore, the magnetic field required for device operation is relatively low (*e*.*g*., 200 to 300 mT), so a small neodymium magnet is strong enough to operate them.

The MECs generated an electromotive force of 130 mV at maximum. The MFETs showed a huge MR effect (503%) at room temperature. Although we still need to overcome some drawbacks related to magnetic field control, switching of the selected cells only, use of liquid electrolyte and so on, these devices exhibited behaviors not observed in conventional electrochemical devices. These unique behaviors can be used to explore novel electrochemical applications based on remote control of electrochemical behavior.

Interfaces and magnetism are two of the few areas that still remain in materials science and physics that have been intensively explored. Deriving useful and novel functions (*e*.*g*., energy storage and switching) from them is important for dealing with the serious problems we are facing in this century (*e*.*g*., energy and resource depletion and the information explosion). Our approach should attract researchers toward development of useful magnetrolytes (*e*.*g*., ionic liquids composed of Fe^3+^ ion, solid magnetrolyte)^32–35^, high surface area electrode materials, and various combinations of the two that will lead to the development of a novel class of electrochemical devices although enhancement of the magnetic field gradient near the electrodes is needed to increase EMF. Investigation of the interactions among the magnetic field, EDL, and magnetic properties (*e*.*g*., spin, magnetization) of materials should be an important research field related to a wide spectrum of magnetic materials.

## Methods

### Fabrication and electrochemical measurement of MEC

MEC was fabricated by placing two electrodes (Au, carbon, or Al) inside a glass square bottle made of chemically inert SiO_2_ glass (10 mm wide; 450 mm high) in a symmetric configuration with an intra-electrode distance of about 9 mm. The electrodes were attached to the inside walls of the bottle with epoxy resin. [Bmim]FeCl_4_ liquid electrolyte (Kanto Kagaku, Japan) was mixed with distilled water in another glass bottle to obtain a solution with the desired concentration (10 to 100%). This solution was then transferred to the glass bottle with electrodes to form the MEC. The effective electrode area was fixed at a 7 × 7 mm by covering the electrode surface with insulating SiO_2_ thin film (deposited by RF sputtering) or epoxy resin. Au and Al plate (Nilaco corp. Japan) was used for the Au and Al electrodes. Carbon thin film on a conducting silicon substrate (MPS Corp. Japan) was used for the carbon electrodes.

Electrochemical measurements of the MEC in a two-terminal configuration were performed using a CompactStat potentiostat/galvanostat/frequency response analyser (Ivium Technologies, Italy) in open circuit and galvanostat modes. A magnetic field was applied to the MEC by applying neodymium magnets with various magnetic flux densities to the outer wall of the glass bottle from the right or left side to apply magnetic field [“ON (right)” and “ON (left)”, respectively]. The magnetic field was removed [“OFF”] by detaching them. The magnetic field profiles inside the MEC were calculated on the basis of a charge model. The magnetic field at the magnet surface was measured using a Hall-effect Tesla meter and used for the profile calculation (see S1). The calculated magnetic field at the electrode/diluted [Bmim]FeCl_4_ interface was used as the magnetic field for each condition.

Optical measurement was performed using a V-7200 UV-Vis-NIR spectrometer (JASCO Corp. Japan) to investigate the optical properties of the 50% diluted [Bmim]FeCl_4_ solution in a magnetic field. The absorbance of the MEC with a magnetic field (*H* = 480 mT) applied from the right side was measured near the two electrodes by attaching a slit with a 2-mm width to limit the light cross-section.

The discharge performance of the MEC [shown in Fig. 2(a)] was measured as follows. First, a magnetic field was applied for 1000 s to charge the MEC. It was then discharged under a constant current of 1 µA, and the voltage variation was monitored. After being discharged, the MEC was charged again using another neodymium magnet that created a different magnetic field. The cycle was repeated several times to investigate the magnetic field dependence of the discharge performance.

### Fabrication and electrochemical measurement of MFET composed of hydrogen-terminated diamond

Hydrogen-terminated diamond thin film was homoepitaxially grown on the surface of an Ib-type high-pressure high-temperature (HPHT) diamond single crystal (100) (Element Six, Luxembourg) using microwave plasma chemical vapour deposition (MPCVD)^24^. The hydrogen (H_2_) and methane (CH_4_) gas fluxes during the deposition process were 1000 and 0.5 sccm, respectively. The deposition temperature was 1213 K. The thickness of the grown epilayer was about 500 nm. A hydrogen-terminated channel (500-*μ*m-long; 800-*μ*m-wide) was fabricated using UV-ozone treatment for 20 minutes at a substrate temperature of 353 K in order to form an insulating oxygen-terminated region outside the channel. Pd, Ti and Au thin films (10, 10, 200 nm thick, respectively) were deposited by electron beam evaporation onto the hydrogen-terminated surface to form source and drain electrodes. Indium shots were used to create electrical connections between the MFET device and two leads made of 10-nm-thick Ti and 200-nm-thick Pt thin films deposited on a sample holder with an insulating SiO_2_-coated silicon substrate. The sample holder and the device (except for the hydrogen-terminated channel) were then covered by epoxy resin for insulation and attached to a glass bottle similar to that used in the MEC.

Electrochemical measurements were performed in air at room temperature using a Keithley 4200-SCS parameter analyer. The drain current variation due to switching of the applied magnetic field was monitored in two-terminal mode at a constant drain voltage of 100 mV.

## Electronic supplementary material

Supplementary information
