# Supplementary material for: Magnetic Control of Magneto-Electrochemical Cell and Electric Double Layer Transistor
Source: Sci Rep. 2017 Sep 5;7:10534. doi: 10.1038/s41598-017-11114-2 (PMC5585326; doi:10.1038/s41598-017-11114-2)
Supplement: Supplementary file 1 — Supplementary information [file 41598_2017_11114_MOESM1_ESM.pdf]

Supplementary information

Magnetic Control of Magneto-electrochemical Cell and Electric Double Layer Transistor

Takashi Tsuchiya[1*], Masataka Imura[2], Yasuo Koide[3], and Kazuya Terabe[1]

1. International Center for Materials Nanoarchitectonics (WPI-MANA), National Institute for Materials Science (NIMS), 1-1 Namiki, Tsukuba, Ibaraki 305-0044, Japan

2. Environment and Energy Materials Division, NIMS, 1-1 Namiki, Tsukuba, Ibaraki 305-0044, Japan

3. Research Network and Facility Services Division, NIMS, 1-2-1 Sengen, Tsukuba, Ibaraki 305-0047, Japan

S1. Magnetic field profile calculation for MEC on basis of charge model

With permanent magnets composed of rare earth elements (*e.g.* neodymium), the magnetization is kept at saturation magnetization in demagnetization curve (*J-H* curve) because the internal magnetic moment is not affected by an external magnetic field. The magnetic field profile near a neodymium magnet can thus be calculated on the basis of a charge model in which the magnetic charge is assumed to be distributed homogeneously on the surface of the magnet.[Ref. S1]

Figure S1 (a) left panel illustrates the calculation condition of the magnetic field profile. The magnetic field profile in one dimension (perpendicular to magnet plane) is given by

$$H(x) = \frac{B\mathrm{r}}{2}\left\{ \tan^{-1}\frac{a\cdot b}{2x\sqrt{4x^2+a^2+b^2}} - \tan^{-1}\frac{a\cdot b}{2(L+x)\sqrt{4(L+x)^2+a^2+b^2}} \right\}$$

(eq. S1-1)

where $H(x)$, B_r, a, b, L, and x are the magnetic field perpendicular to the magnet plane (T), the residual magnetic flux density of the magnet (T), the height of the magnet (mm), the width of the magnet (mm), the length of the magnet (mm), and the distance from the magnet plane (mm), respectively.

Figure S1 (a) right panels show the magnetic field (upper panel) and magnetic field gradient (lower panel) profiles inside the MEC for various neodymium magnets calculated on the basis of the charge model. The B_r values for the magnets used were 0.547, 0.450, 0.429, 0.372, 0.325, 0.285, 0.219, 0.165, and 0.087 T. The magnetic field gradient is relatively steep within about 3 to 4 mm of the electrode surface on the magnet side. The magnetic field dependence of various characteristics of the MFEC are discussed below with respect to the magnetic field at the electrode/diluted [Bmim]$FeCl_4$ interface.

When a magnet is attached to the MEC from the right side, the magnetic field gradually decays from the electrode/diluted [Bmim]$FeCl_4$ interface on the right side to that on the left side and vice versa. Three situations for magnetic field application, ON (Left), ON (Right), and OFF, and the corresponding magnetic field gradient profiles are shown in Figs. S1(b) and (c), illustrating that the direction of [Bmim]$FeCl_4$ attraction is switched from right to left by changing the magnet position, "magnetic field switching (left/right)". Although magnetic field switching (ON/OFF) causes EMF

switching, as clearly seen in Fig. 2(a), magnetic field switching (left/right) creates a significant difference in EMF, as will be discussed in Figs. S2. This principle is thus used in EMF switching in the MEC [shown in Fig. 2] and drain current switching in the MFET [shown in Figs. 4 (b, c)].

In a magnetic field, the $FeCl_4^-$ ions, which are sensitive to a magnetic field due to their strong paramagnetic characteristics, start to move. This movement generates a local electric field, which causes the $[Bmim]^+$ ions, which are insensitive to a magnetic field, to move in the same direction. This process can be classified as ambipolar diffusion.

[Ref. S1] E. P. Furlani. Permanent Magnet and Electromechanical Devices: materials, analysis and applications. Academic Press, London, 6th edition, 2001.

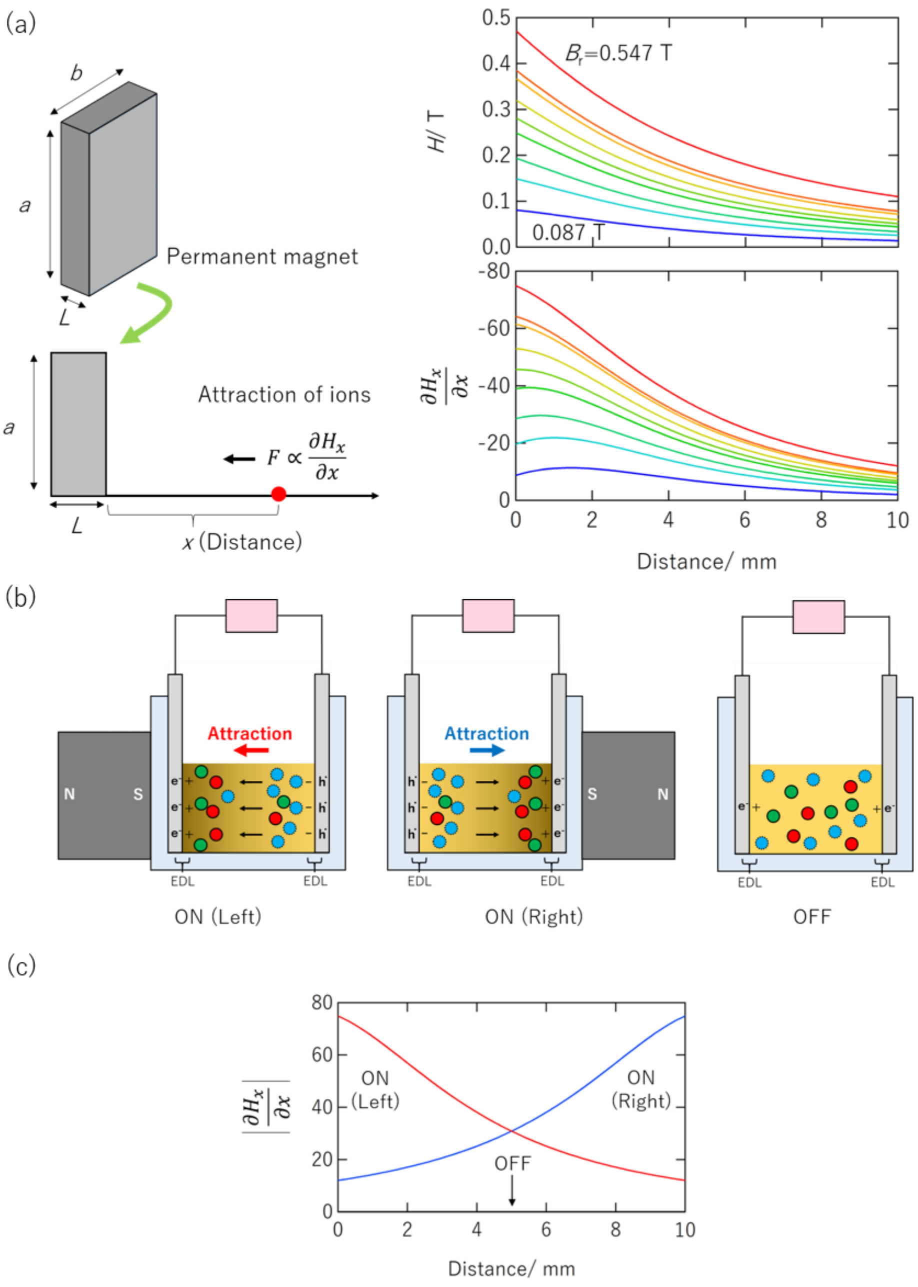

Figure S1(a, left) Illustration of calculation conditions for magnetic field profile. (a, right) Magnetic field (upper) and magnetic field gradient (lower) profiles inside MEC. (b) Three situations for magnet field application. (c) Magnetic field gradient profile for three conditions. The value is shown as an absolute value.

S2. Repeatability of EMF modulation and investigation of behaviour with reference electrode

Figure S2 (a) shows the repeatability of the EMF modulation in response to magnetic field switching (480 mT) caused by switching the magnet position between the left and right sides of the MEC. The switching technique is described in S1. The device showed good repeatability for 20 switching cycles without any significant degradation in the EMF property. On the basis of the observations shown in Figs. 2(a) and S2(a), we concluded that a high [Bmim]$FeCl_4$ concentration near one electrode makes the potential of that electrode negative (*i.e.* the EDL dopes additional electrons in the electrode) and vice versa. This was confirmed in an additional experiment using an MEC with a reference electrode.

Figure S. 2(b) shows the variation in EMF for an MEC with an Au working electrode, an Au counter electrode, and an Ag/AgCl (silver/silver chloride electrode, SSE) reference electrode. The potential of the working electrode without a magnetic field was 590 mV vs. SSE (E_{off}). When a magnetic field was applied to the cell from the working electrode (right) side, the potential dropped to 520 mV vs. SSE ($E_{\mathrm{on,\ right}}$). In contrast, when a magnetic field was applied to the counter electrode (left) side, the potential of the working electrode increased to 640 mV vs. SSE ($E_{\mathrm{on,\ left}}$). The potential difference between the two conditions, $E_{\mathrm{on,\ left}}$ - $E_{\mathrm{on,\ right}}$, corresponds to the EMF of the EDLC observed in Fig. 2(a), namely E_{left} - E_{right}.

$$\Delta E = E_{\mathrm{on,\ left}} - E_{\mathrm{on,\ right}} = E_{\mathrm{left}} - E_{\mathrm{right}} = \mathrm{EMF} \qquad \text{(eq. S2-1)}$$

Therefore, the EMF behaviour illustrated in Fig. S2(b) agrees well with those shown in Figs 2(a) and S2(a). That is, a high and low concentration of [Bmim]$FeCl_4$ induce negative and positive charges into the electrodes, respectively.

While the EMF saturated to $E_{\mathrm{on,\ right}}$ in 500 to 600 s, the EMF transition to $E_{\mathrm{on,\ left}}$ was not completely finished for more than 5000 s. The significant difference in the relaxation behaviour may be related to the ionic diffusion environment in high and low concentration [Bmim]$FeCl_4$ regions generated by the applied magnetic field. The very slow kinetics of the EMF transition to $E_{\mathrm{on,\ left}}$ indicates that there were sufficient water molecules in the low concentration [Bmim]$FeCl_4$ region to slow the ionic (ambipolar) diffusion in a magnetic field. The physical properties of the solvent (including polarizability and magnetism) can thus affect the diffusion process.

To investigate effect of H polarity (S polar or N polar) on electrode potential and EDL charge behavior, $E_{\mathrm{on,\ right}}$ was measured by applying H using S polar or N polar of the magnet (480 mT). Figure S.2(c) shows $E_{\mathrm{on,\ right}}$ observed 2000 s after H application using S polar (1st, 3rd, 5th cycle) or N polar (2nd, 4th, 6th cycle). This result indicated that H polarity gives no significant effect on

electrode potential and EDL charge behavior in the present case. While this result indicates that the *H* reversal effect is small in our case, it is still possible that the *H* reversal effect on EMF is much significant in other cases.

Three-terminal electrochemical measurements [shown in Fig. S2(b) and (c)] were performed using PARSTAT 2273 potentiostat/galvanostat/frequency response analyser (Princeton Applied Research, USA) in open circuit mode. An Ag/AgCl (silver/silver chloride) reference electrode was immersed in an MEC with Au/Au electrodes to monitor the electrode potential of the Au working electrode with respect to the potential of the Ag/AgCl reference electrode, which is typically 0.222 V vs. standard hydrogen electrode (SHE) under $[Cl^-]$=1.00 M conditions.

(a)

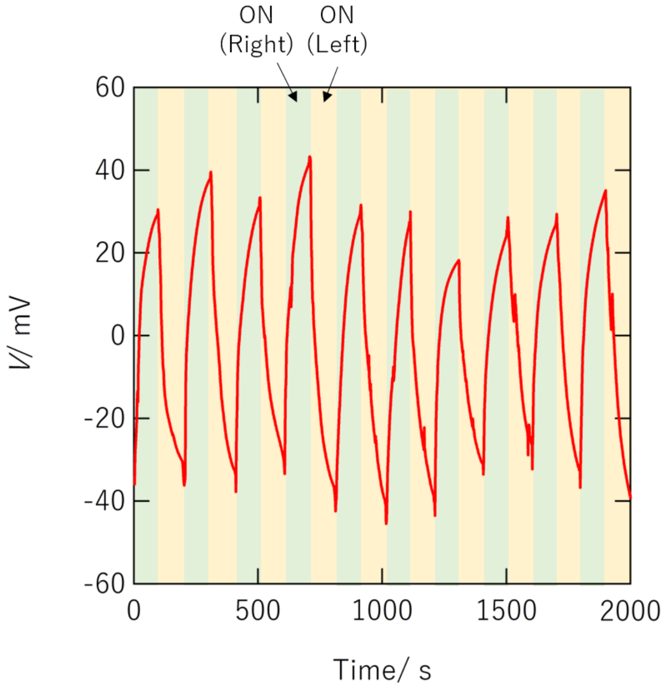

Figure S2(a) Repeatability of EMF modulation in response to switching magnetic field (480 mT) on and off.

(b)

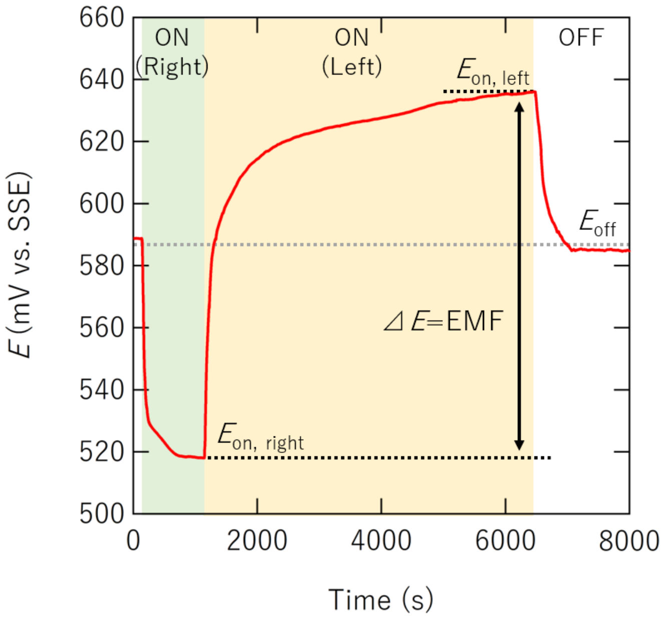

(c)

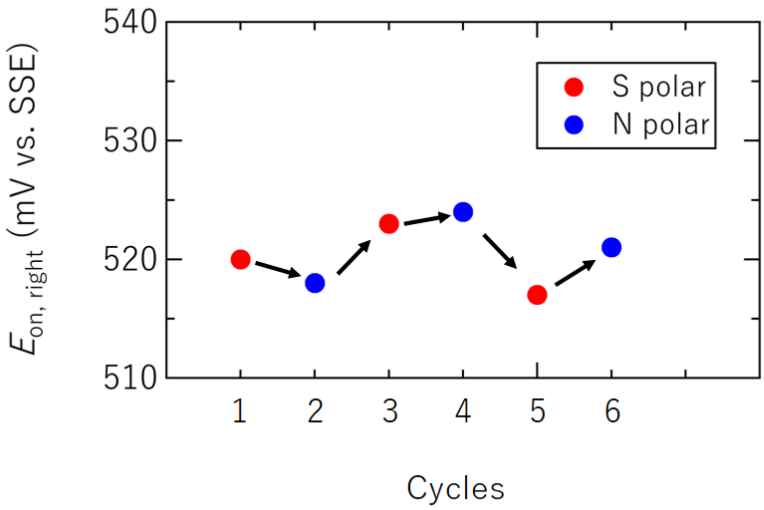

Figure S2 (b) Variation in OCV of MEC with Au working electrode, Au counter electrode, and SSE reference electrode. (c) $E_{\mathrm{on,\ right}}$ achieved by H application using S polar or N polar. While H was applied using S polar of the magnet in 1st, 3rd, and 5th cycles, it was applied using N polar of the magnet in 2nd, 4th, and 6th cycles.

S3. Noisy and unstable OCV variation with aluminum electrodes

Figure S3 shows the variation in OCV of an MEC with two Al electrodes (Al/Al) and 10% diluted [Bmim]$FeCl_4$ solution. The variation was significant even without magnetic field application. This noisy and unstable behaviour indicates the occurrence of electrochemical reactions at the electrode interface. When a magnetic field was applied to the MFEC from the right side, the OCV increased to -140 mV. The applied magnetic field greatly increased the [Bmim]$FeCl_4$ concentration near the right electrode relative to that near the left electrode, creating an EMF between the two electrodes. The polarity of the EMF was opposite that for carbon/carbon and Au/Au MFECs, indicating that the origin of the EMF differed from EDL. Considering that the Al electrodes strongly reacted with the Cl^- ions in the [Bmim]$FeCl_4$ resulting in $AlCl_3$ generation on the surface of the Al electrodes (as will be shown in S4), we concluded that the EMF in the cell originated from the redox reaction of Al.

After the OCV reached a maximum, the EMF slightly decreased from -140 to 105 mV. Further repetition of the cycles caused degradation of the electrodes, resulting in very small EMF switching. This behaviour also indicates that the electrochemical reaction contributed to the EMF. It verifies our assumption.

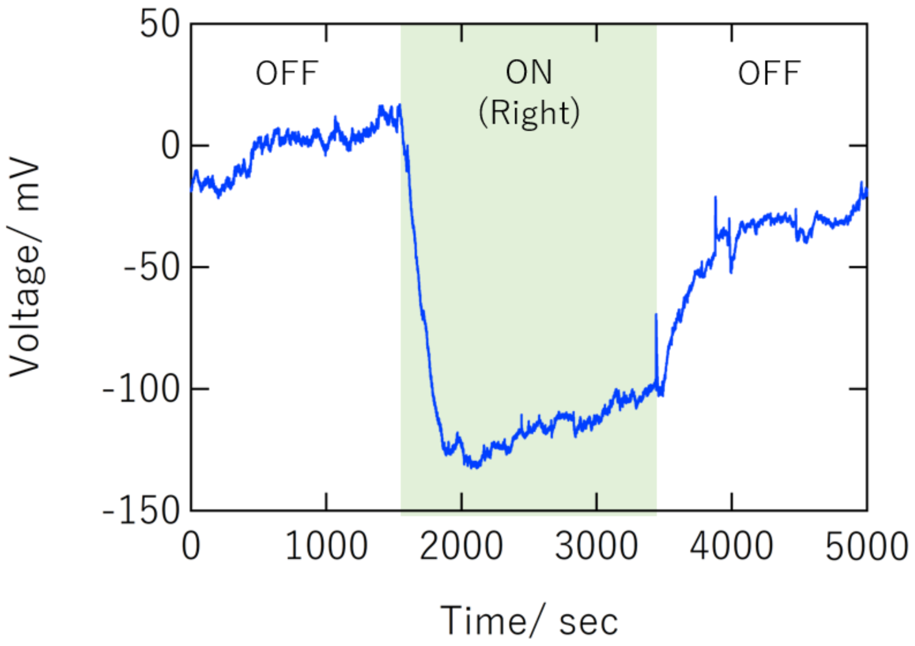

Figure S3. Variation in OCV of MEC with two Al electrodes (Al/Al) and 10% diluted [Bmim]$FeCl_4$ solution.

S4. Generation of aluminium chloride on surface of Al electrode

Since aluminium (Al) is highly reactive metal, reaction S.4 proceeds in the presence of Cl^- ions in aqueous solution, resulting in the generation of aluminium chloride ($AlCl_3$) on the surface of an Al electrode.

$$\mathrm{Al} + 3\mathrm{Cl}^- \rightarrow \mathrm{AlCl}_3 + 3e' \qquad \text{[S4-a]}$$

Figure S4 shows the x-ray diffraction pattern (XRD) of an Al electrode measured after MEC operation with [Bmim]$FeCl_4$. The (002), (131), and (-331) peaks of $AlCl_3$ were clearly observed in addition to Al (220), Al (211), and Al_2O_3 (113) peaks as well as several unknown peaks. The weak peaks reflect the low crystalline nature of $AlCl_3$ generated by the electrochemical reaction [S4] at low temperature such as room temperature. Energy dispersive x-ray spectrometry revealed a large number of Cl ions in the $AlCl_3$ surface layer.

Although the electrochemical reaction (S4) under magnetic field conditions caused EMF as well as EDL charging in the carbon/carbon and Au/Au cells (shown in Fig. 2), the EMF is originated from electron energy, or chemical potential of electron ($\mu_{e'}$), difference between the two Al electrodes due to the different equilibrium states of the reaction.

In the equilibrium of reaction (S3), the electrode potential is expressed by the Nernst equation:

$$E = E^0 - \frac{RT}{3F} \ln \frac{a_{\mathrm{Al}} \cdot \left[\mathrm{Cl}^-\right]^3}{a_{\mathrm{AlCl_3}}}, \qquad \text{(eq. S4-1)}$$

where E^0 , a_{Al}, $a_{\mathrm{AlCl_3}}$, and $\left[\mathrm{Cl}^-\right]$ are the standard electrode potential of the reaction, the activity of Al, the activity of $AlCl_3$, and the concentration of Cl^-, respectively. Given that a_{Al} and $a_{\mathrm{AlCl_3}}$ are unity, this equation can be simplified to a function of $\left[\mathrm{Cl}^-\right]$:

$$E = E^0 - \frac{RT}{F} \ln\left[\mathrm{Cl}^-\right]. \qquad \text{(eq. S4-2)}$$

Although it is difficult to estimate the electrode potential merely with eq. S4-2 because $\left[\mathrm{Cl}^-\right]$ is strongly affected by the following dissociation equilibrium, which can differ between two electrode interfaces, the OCV variation shown in Fig. S3 can be qualitatively understood based on eq. S4-2.

$$\mathrm{FeCl}_4^- \rightarrow \mathrm{FeCl}_3 + \mathrm{Cl}^- \qquad \text{[S4-b]}$$

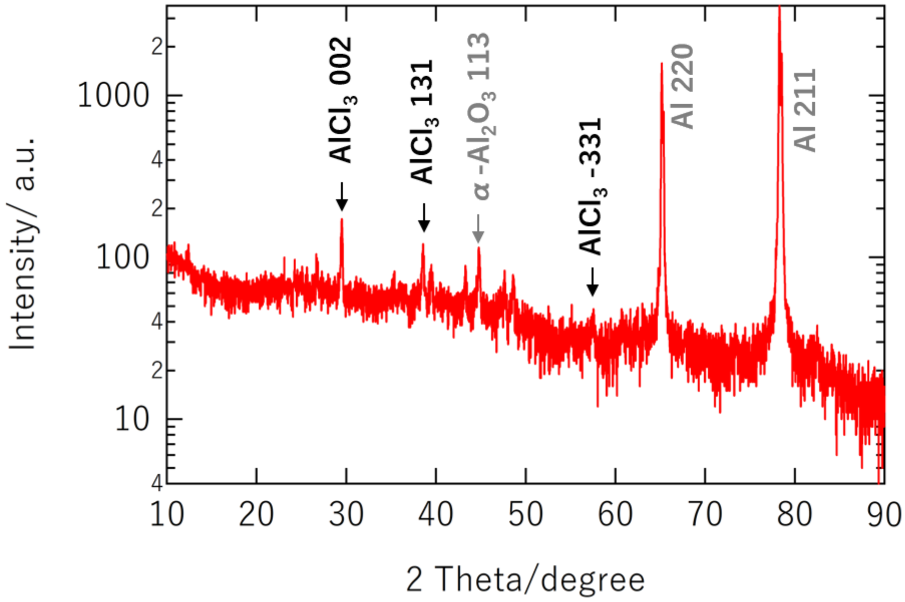

Figure S4. XRD pattern of Al electrode measured after operation of MEC containing [Bmim]$FeCl_4$.

S5. Origin of Q in discharge

To investigate the origin of the large Q in the discharge, two additional experiments were performed. The first was done to investigate the dependence of the Q on an effective electrode area of MEC. An MEC was fabricated with a reduced effective electrode area of 3 × 3 mm (～18% that of the original one (7 × 7 mm). The MEC was discharged using the same procedure as in the experiments with H=480 mT [Fig. 3(a)]. The observed Q (13 mC) is plotted in Fig. S5(a) along with that of the original device (16 mC) for reference. If the Q originates from the EDL charge, it should be proportional to the effective electrode area, and the two values should thus be on a straight line. However, the Q for the MEC with the reduced effective electrode area was 4.5 times larger than that expected from the direct proportional relationship. This demonstrates that the Q does not originate from the EDL charge.

The other additional experiment was performed to investigate the dependence of Q on the volume of electrolyte in the MEC. The total volume of electrolyte liquid (50% diluted [Bmim]$FeCl_4$) in the MEC was doubled to 2 mL. The MEC was discharged using the same procedure as in the experiments with H=480 mT [Fig. 3(a)]. The observed Q (27 mC) is plotted in Fig. S5(b) along with that of the original device (16 mC) for reference. If the Q originate from the EDL charge, it should be insensitive to the electrolyte volume. However, the Q strongly depended on the volume. This indicates that the Q did not originate from the EDL charge but from the electrochemical (Faradaic) charge.

Note that, in principle, the EDL charge of a concentration cell is modulated if the concentration of electrolyte is varied regardless of the method (electric field, magnetic field). In the MEC, the EDL charge is thus modulated during operation. But the Q that originated from the EDL was far smaller than the Q that originated from the electrochemical reactions accompanied by modulation of the ion concentration. That is why we observed a large Q in the MEC.

(a)

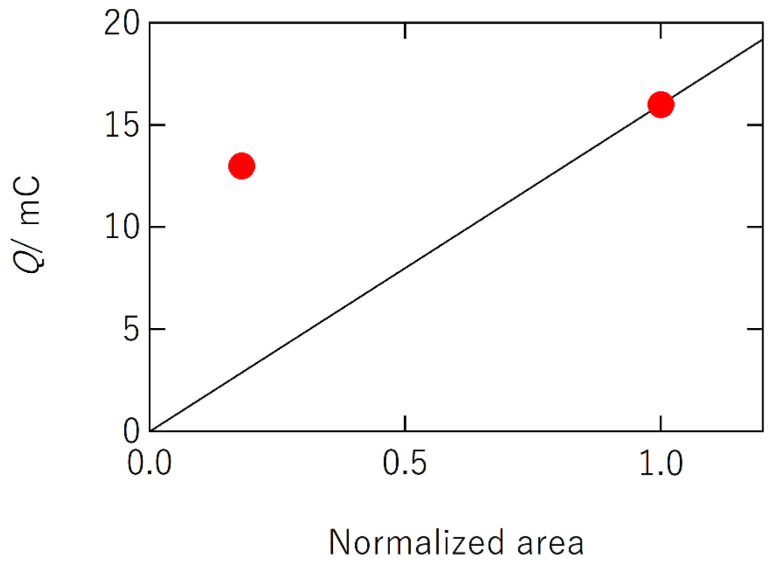

(b)

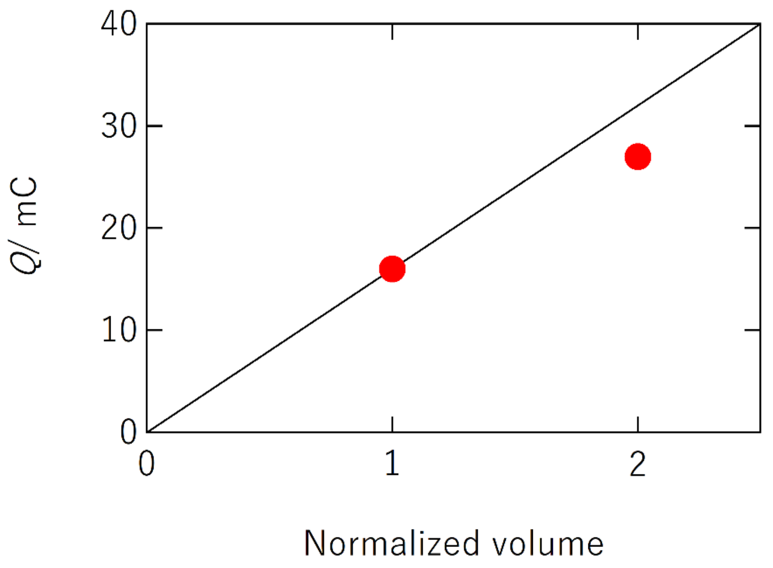

Figure S5. Dependence of Q in discharge on (a) normalized effective electrode area and (b) normalized volume of electrolyte.

S6. Origin of EMF in MEC and MFET

The EMF behaviour of the MEC, which is modulated by varying the concentration of solute ([Bmim]$FeCl_4$) in the liquid electrolyte, is similar to that of electrochemical concentration cells as discussed on the basis of Nernst equation [equation (2')]. In addition to the EDL, the junction potential near the interface between the high and low concentration regions can modulate the EMF. The junction potential (V_{jp}) is expressed by

$$V_{\mathrm{jp}} = \left(t^{+} - t^{-}\right)\frac{RT}{F}\ln\frac{a_1}{a_2}, \qquad \text{(eq. S6)}$$

where t^+, t^-, a_1, and a_2 are the transport number of cations, that of anions, the activity of the solute near the left electrode, and that near the right electrode, respectively. Activity can be further transformed into the product of the activity coefficient, γ, and the concentration, c: $a=\gamma c$. Given that γ is unity, the activity of the solute (a) can be replaced by the solute concentration (c).

Figures S6 (a, b, c) illustrate the potential profiles in three situations: (a) initial state, (b) EMF generation without junction potential, and (c) EMF generation with significant contribution from junction potential. While EMF generation without junction potential [Fig. S. 6(b)] produces significant modulation of the charges at the EDL, EMF generation with significant contribution from junction potential [Fig. S. 6(c)] does not affect EDL charging.

To clarify the actual situation in our study, we investigated a contribution from the junction potential to the observed EMF. Figure S. 6(d) shows the variation in EMF for an MEC with an Au working electrode, an Au counter electrode, and an SSE reference electrode, which was located at the left side wall in the initial condition. The potential of the working electrode without a magnetic field was about 593 mV vs. SSE (E_{off}). When a magnetic field was applied to the cell from the working electrode (right) side, the potential dropped to 520 mV vs. SSE ($E_{\mathrm{on,\ right}}$). This behavior was similar to that observed in Fig. S2(b). Then, the location of the reference electrode was changed to right side wall (almost touched to the working electrode) as indicated by black arrow. The EMF was slightly shifted to positive direction (approximately 2 mV). The shift corresponds to V_{jp} discussed above. Based on the result, the contribution from the junction potential is an order of several %, and the actual situation is close to Fig. S6(b). The small V_{jp} may be due to small t^+-t^- in eq. S6.

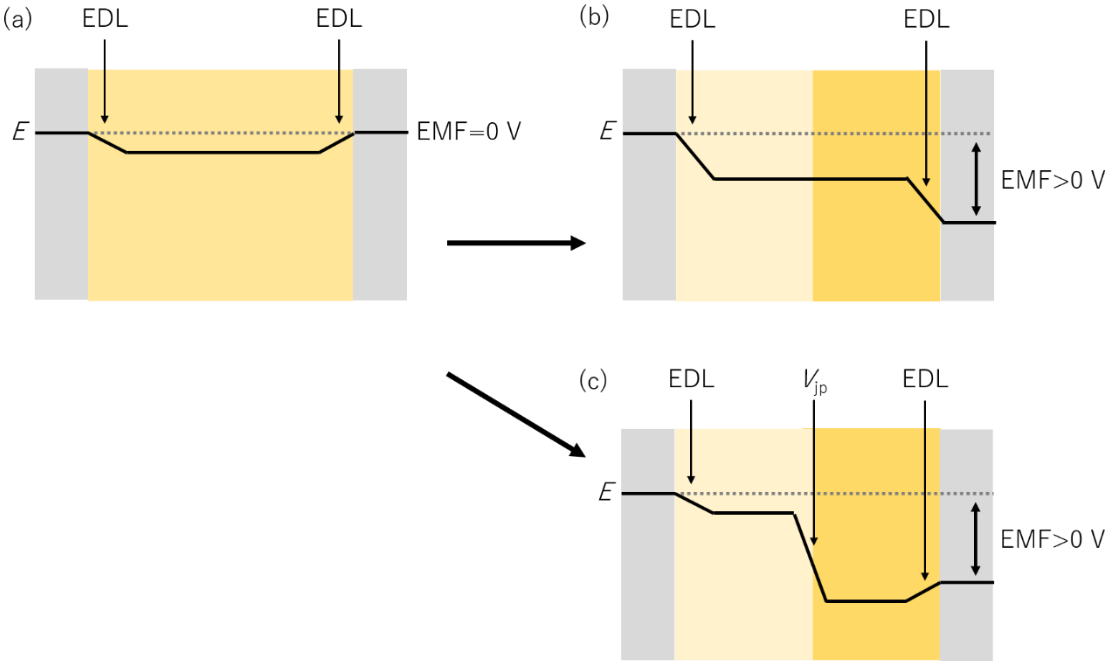

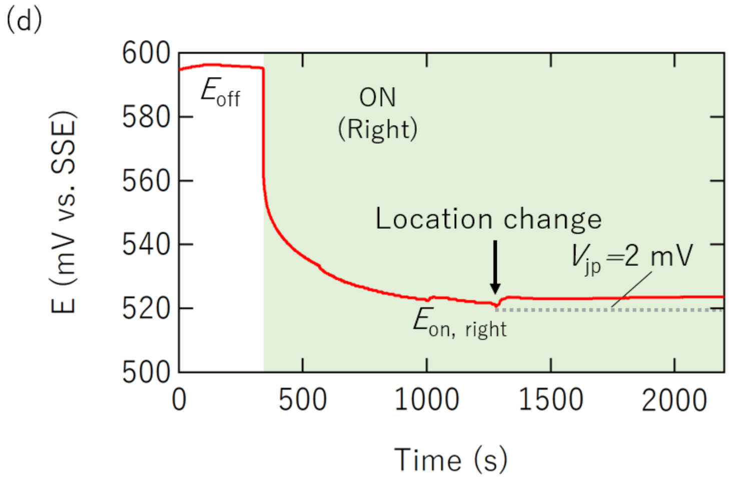

Figure S6. Illustrations of potential profile in three situations: (a) initial state, (b) EMF generation without junction potential, and (c) EMF generation with significant contribution from junction potential. (d) Variation in OCV of MEC with Au working electrode, Au counter electrode, and SSE reference electrode. The location of the reference electrode was changed from left side wall to right side wall at the point indicated by the black arrow.

S7. XRD pattern of homoepitaxially grown hydrogen-terminated diamond thin film on diamond single crystal

Figure S7 shows the XRD pattern of homoepitaxially grown hydrogen-terminated diamond thin film on diamond single crystal. Only (400) diffraction at around 120° was observed over the whole range except for the kβ line at 103°, indicating excellent quality of the hydrogen-terminated diamond thin film with (100) orientation.

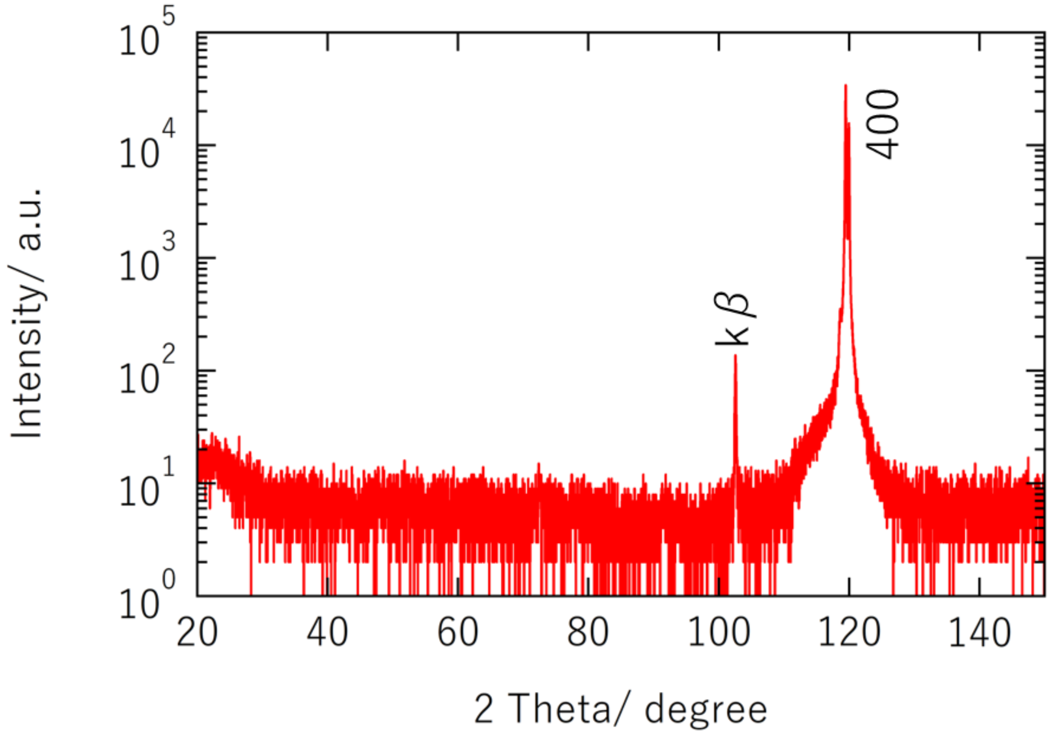

Figure S7. XRD pattern of homoepitaxially grown hydrogen-terminated diamond thin film on diamond single crystal

S8. Investigation of background magnetoresistance effect in hydrogen terminated diamond channel of MFET

The magnetoresistance (MR) effect is defined as electric conductivity modulation induced by a magnetic field. The mechanisms of this effect has been widely varied from a basic one (Lorentz force applied to electrons) to a more sophisticated one (spin-dependent tunnel current in the vicinity of a tunnel junction).

To rule out a possible contribution from background MR effect (intrinsic for hydrogen-terminated diamond) to electric conductivity modulation in the MFET, we investigated the drain current behaviour of a hydrogen-terminated diamond channel in a magnetic field prior to the MFET operation shown in Fig. 4. Figure S8(a) shows the experimental setup. A hydrogen-terminated diamond-based transistor was set inside a glass square bottle similar to MFET operation but without [Bmim]$FeCl_4$. Figure S8(b) shows the drain current (i_D) variation when the magnetic field was changed by attaching a neodymium magnet (480 mT) to the outside of the bottle. No i_D modulation was observed, indicating that background MR has little effect in the present magnetic field range at room temperature.

(a)

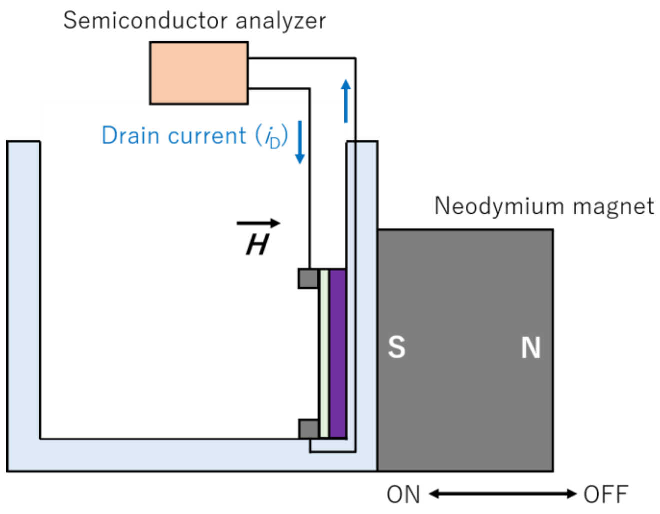

(b)

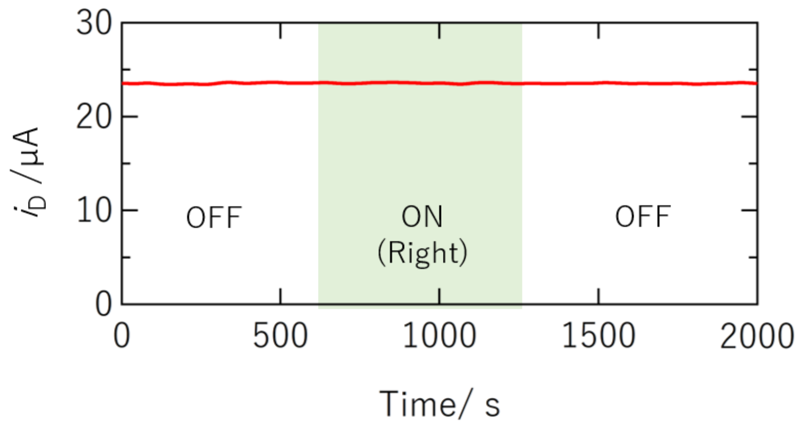

Figure S8(a) Experimental setup for MR measurement. (b) i_D variation when magnetic field was changed by attaching a neodymium magnet (480 mT) to outside of bottle.
